# Living with glioblastoma — the need for integrated support based on experiences of chaos, loss of autonomy, and isolation in both patients and their relatives

**DOI:** 10.1007/s00520-024-08801-y

**Published:** 2024-08-21

**Authors:** Pernilla Ståhl, Ingela Henoch, Bertil Rydenhag, Anja Smits, Anneli Ozanne

**Affiliations:** 1 Institute of Health and Care Sciences, Sahlgrenska Academy, University of Gothenburg, Box 100, 40530 Gothenburg, SE Sweden; 2 Department of Neurosurgery, Sahlgrenska University Hospital, Gothenburg, Sweden; 3 Department of Clinical Neuroscience, Institute of Neuroscience and Physiology, Sahlgrenska Academy, University of Gothenburg, Gothenburg, Sweden; 4 Department of Neurology, Sahlgrenska University Hospital, Gothenburg, Sweden

**Keywords:** Brain tumor, Family, Glioblastoma, Patients, Relatives, Support

## Abstract

**Purpose:**

The aim of this study was to investigate the experiences of living with glioblastoma from the perspective of patients themselves as well as their closest relatives, focusing on the changes in the life situation and the need for support.

**Methods:**

Twenty-two semi-structured interviews were conducted with 12 patients (mean age 61 years, 7 male, 5 female) and 10 relatives (mean age 56 years, 3 male, 7 female). The relatives comprised of partners (*n* = 7), child (*n* = 1), sister (*n* = 1), or friend (*n* = 1). Questions focused on changes in the life situation and support needed to face these changes. Data was analyzed using inductive qualitative content analysis (QCA).

**Results:**

Living with glioblastoma dramatically changes the lives of both patients and relatives. Cognitive symptoms (e.g., speech and memory disturbances), deterioration of physical function (e.g., paresis), and psychological function (e.g., behavioral changes, anxiety) can lead to impaired family dynamics, social isolation, and fear of the future. Support from other family members, friends, and healthcare professionals is crucial. Timely, tangible, and easily available support from the healthcare system the entire disease trajectory is sought after, enabling individualized care with emotional support, clearer information, and faster feedback.

**Conclusion:**

The changes in life situations faced by patients with glioblastoma and their closest relatives are dramatic and underline the importance of providing integrated care throughout the entire healthcare continuum, encompassing specialist neuro-oncological care, municipal support, and palliative care. Individualized support for both patients and relatives can enhance the sense of safety amid the chaos in their life situation.

**Supplementary Information:**

The online version contains supplementary material available at 10.1007/s00520-024-08801-y.

## Introduction

Glioblastomas are the most common and most malignant primary brain tumors [1], originating from glial cells and characterized by the absence of IDH mutations (IDH-wildtype) [2]. The average age at diagnosis is about 65 years [1]. Multimodal treatment consists of a combination of surgery, radiotherapy, and chemotherapy [3, 4]. In addition, symptomatic treatment with corticosteroids, analgesic, and/or antiepileptic drugs [5, 6] is frequently used.

Patients with glioblastoma face a poor prognosis [3], with 8–25% surviving beyond 2 years [7, 8]. Disease- and treatment-related symptoms consist of a variety of cognitive problems, focal neurological deficits, epileptic seizures, and signs of increased intracranial pressure, such as headache and nausea [6, 9, 10]. Along with progressive disease, patients may become more apathetic and somnolent, experience negatively affected mood, and lose their autonomy [6, 10]. These symptoms all lead to a higher degree of dependence, changed family roles, and a risk of becoming isolated. Relatives may feel obliged to take greater responsibility as caregivers, which further encroaches on everyday life [11]. The seriousness of the disease and its consequences on daily life underscore the importance of investigating the life situation and support of both patients and their relatives.

The mental health of patients and relatives is affected throughout the disease trajectory [12–14]. There is a relationship between the patients’ and relatives’ HRQoL and psychological symptoms within the families [13]. However, the support given to relatives increases the patients’ HRQoL [15]. Informal, emotional, and practical support from family and friends is of importance for patients with brain tumors [16, 17]. Relatives may also feel forced to care for the patient without access to support or information themselves [18]. Others may wish to become more involved in their care, thereby feeling supported and experiencing greater well-being [19]. To conclude, providing optimal support is not straight forward and although informal support from family and friends is perceived as important and a great asset by patients, it can also be perceived as a burden [16, 20].

From a healthcare perspective, patients with high-grade glioma need a multidisciplinary team (e.g., physicians, nurses, physiotherapists, occupational therapists, neuropsychologists, and social workers) that can identify the type of support needed [19–21]. Previous studies have highlighted an unmet need for emotional and mental support in patients with brain tumors and their relatives [20, 22, 23]. Still, there is a knowledge gap regarding support for patients with glioblastoma and their relatives, as the course of disease in glioblastoma differs from other brain tumor diagnoses in terms of severity, but also from one patient to another [24]. There is a dearth of information regarding the experiential aspects and optimal support for patients with glioblastoma and their relatives, viewed from an integrative care perspective and covering the entire trajectory of the disease. Instead, various care providers base their service on their individual organizational structures rather than collaboratively addressing the needs of patients and their families. The aim of this study was to investigate the experiences of living with glioblastoma from the perspective of patients themselves as well as their closest relatives, focusing on the changes in the life situation and the need of support.

## Method

### Study design

This study has a qualitative inductive approach and includes patients with IDH-wildtype glioblastoma [2] and their closest relatives. An inductive approach was employed to ensure that data collection and analysis were conducted as unconditionally as possible without being influenced by theories or previous knowledge about the topic.

### Participants

The inclusion criteria for the group of patients were 18 years or older and diagnosed with IDH-wildtype glioblastoma. At least 2 months had passed since surgery, to allow patients to recover from surgery and start oncological treatment. Exclusion criteria were the patient’s inability to participate in the interview due to severe cognitive impairment or severe speech difficulties. Relatives were primarily selected based on the patient’s wishes. Inclusion criteria were at least 18 years old; no exclusion criteria based on cognitive or language performance were used for the group of relatives. Inclusion and exclusion criteria were in accordance with the application approved by the Ethics Committee. Eligible candidates (*n* = 54) were identified by clinical nurses at multi-disciplinary neuro-oncological conferences at the University Hospital in Gothenburg, Sweden, where all patients presenting with suspected primary brain tumors in the Västra Götaland healthcare region (2.1 million inhabitants) are discussed.

**Fig. 1:**
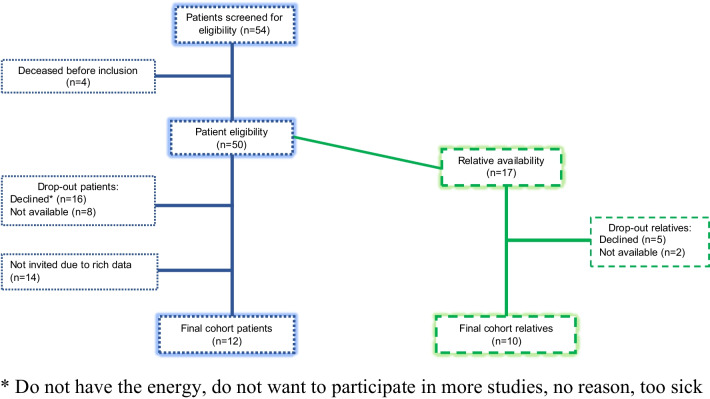
Recruitment flow chart. *Do not have the energy, do not want to participate in more studies, no reason, too sick

After excluding not eligible patients and drop-outs, 50 patients were eligible to participate in the study. Maximum variation sampling [25], encompassing diverse demographics such as age, gender, and duration since diagnosis, was employed to capture a broad spectrum of perspectives. Data collection ended after including 12 patients and 10 relatives (Fig. 1), since the data was considered sufficiently rich, based on a qualitative approach [26]. The relatives were chosen by the patients and defined as their closest relative, i.e., partner (*n* = 7), child (*n* = 1), sister (*n* = 1), and close friend (*n* = 1). Demographic data are presented in Table 1.

**Table 1:** Participant demographics and interview characteristics

	Patients	Relatives
Gender male/female	7/5	3/7
Age yrs *mean (range)*	61 (32–78)	56 (31–67)
Time from diagnosis		
* 2–5 months*	2	0
* 6–11 months*	4	4
* 1–1.5 years*	1	1
* 1.5–2 years*	1	2
> *2 years*	4	3
Patients cohabiting	9	-
Patients living alone	3	-
Relative sharing housing with the patient yes/no	-	7/3
Relation to patient		
* Partner*	-	7
* Child*	-	1
* Sister*	-	1
* Close friend*	-	1
Type of interview		
* Face to face*	3	1
* Telephone*	8	6
* Video*	1	3
Interview length (minutes)		
Mean (range)	51 (30–74)	40 (16–72)

### Data collection

Data collection for the individual interviews occurred between March 2022 and December 2022. Potential participants were contacted via phone by the first author, informed about the study, and given the opportunity to ask questions. Thereafter, written information about the study and consent forms were sent by post to those who had expressed interest. Upon receiving signed consent, participants were contacted again by phone to schedule an interview and to decide whether the interview would be conducted via phone, video, or in person. On this occasion, participants were also given a further opportunity to ask questions. A semi-structured interview guide (Supplementary material 1) with open-ended questions was developed, using an inductive approach, aimed at addressing the research objectives as unconditionally as possible. Two pilot interviews, one with a patient and one with a relative, were conducted, leading to a few adjustments to the guide. Interviews were conducted in person, by telephone, or by video, based on the participant’s preference (Table 1).

Based on the repetition of content in interview responses and the absence of new information, the data was considered sufficiently rich following interviews with 12 patients and 10 relatives, in accordance with descriptions on how to handle rich data in qualitative studies [26]. The interviews were audio-recorded and transcribed verbatim. The interviews were transcribed by the first author (PS) (*n* = 10) and a transcription service (*n* = 12). PS double-checked that the content of the text files was identical to the audio files and anonymized.

### Data analysis

Data were analyzed with inductive qualitative content analysis (QCA), based on a structured and systematic analysis of written or verbal communication [27, 28]. QCA is suitable for analyzing both manifest and latent content, aligning with the purpose and nature of the data. This approach allows for both descriptive and interpretive analyses. QCA is also suitable for comparing similarities and differences within the data, which was considered particularly relevant given the study’s inclusion of both patients and relatives [28, 27, 29]. The interviews were read through several times by the authors PS and AO before the text was divided into meaning units and condensed with codes by PS. The meaning units and codes were discussed by PS and AO until a consensus was found. The codes were then sorted according to similarities and differences and abstracted into subthemes by PS. These were discussed and revised together by PS, IH, and AO. Finally, the subthemes were abstracted into descriptive themes, representing the manifest and latent content by PS, IH, and AO [27, 29, 30]. To increase trustworthiness, continuous reconciliations were conducted with PS, IH, and AO, and authors went back and forth from parts to the whole of the text. All research group authors participated in final analysis discussions, achieving consensus on subthemes and themes to strengthen trustworthiness further. The analysis was performed in NVivo version 13 [31].

## Results

The results are showing experiences of living with glioblastoma from the perspective of patients themselves as well as their closest relatives, focusing on the changes in the life situation and the need for support. Both patients and their relatives were affected by the disease and the support given. Although partly satisfied with the support, they received from the health care professionals (HCP), they also experienced shortcomings, and opportunities for improvement have emerged. The themes “Hovering between living in chaos and finding strategies in daily life” and “Searching for outside assistance to attain safety in chaos” with subthemes and selected quotes are described in Table 2 and the text below. Table 3 summarizes the potential development of support based on the results from each subtheme.

**Table 2:** Themes, subthemes, and patient and relative quotes

Main theme	**Hovering between living in chaos and finding strategies in daily life**
Subtheme	**Living in uncertainty**
Quotes	“Yes, that’s what they’ve told me, that I’ll gradually become like a helpless package, so this is just the beginning, I’ll lose everything, but it will probably happen gradually so you… I might not even be so aware of it.” (Patient 2) “The thing is … the second time … I have taken various medications for my heart, and I was not supposed to bring my own medications; they were supposed to distribute them, and it has been chaos at the hospital. Even previously when they distributed it, it was chaos. So, I take one medication in the morning and one in the evening, you know, it was total chaos, they don’t understand that.” [the patient describes a chaotic situation in a period of uncertainty] (Patient 6) “It was – I mean the first epileptic seizure she had – it was really a near-death experience for me. So, it was an absolutely terrible experience, I didn’t know if she was going to die, I didn’t know what was happening. So, it was very, very difficult to see her in that condition. It was the same on the other occasions, but then it became more of a sadness, that it had to happen again. But I didn’t feel insecure about the process itself because it took about half an hour before she had kind of recovered a bit or regained her composure, then she was very tired for the rest of the day, so to speak. But it was probably the worst thing I’ve ever experienced, actually.” (Relative 6) “Everything has…Of course I don’t have any…I don’t dare to believe, I don’t know how long our future is. Or I know that we have a limited future, and that anxiety is always there. Everything has changed, you know.” (Relative 8)
Subtheme	**Living with cognitive consequences**
	“Yes, yes, it’s getting harder for me. I can, like, uhh, in my head, see how I should do and plan things, but I haven’t the energy for the actual execution. Oh, like some things, very little gets done.” (Patient 3) “And then also…. auditory images or things like…a lot of people in a room talking and stuff, then she… the input gets too much.” (Relative 7)
Subtheme	**Living in isolation**
Quotes	“Yes, for example, I’ve become especially more cautious in socialising with my grandchildren, I have quite a few, you know, so if they are sick, I’ve had to be more careful, and they said that in certain situations, there was a higher risk of me getting infected, I had a weakened immune system. So I was going to say it has been a bit disappointing not meeting others like that. I… now I’ve seen them a bit more, but not at all as I did before, so that has been a part of it.” (Patient 5) “I get like, I should work, because I need something else, [cries] something that is normal in my life, and then [cries] I have needed to work, and now I can’t do it as much, it’s not possible… And work has probably been the only place where I could disconnect, and rest my mind, and think that I am still a person with skills, that I can still do something.” (Relative 8)
Subtheme	**Managing the new life situation**
Quotes	“Yes, I… So far, I take care of the laundry room myself, because there you can hold on to the walls and such, because it’s not that spacious, but you… there’s always a wall that you can sort of grab onto, right, so that for now, I do the laundry myself. I’ve stopped, stopped [laughs] I’ve stopped hanging things in some drying room, because I can’t be bothered, but I tumble dry everything so that it gets really wrinkled, but it gets dry anyway, dry and warm. And yes, it is done … what … well, only once or twice a month, so there isn’t so much washing here, but I realise I won’t be able to keep doing it forever, but … but I can still do it now.” (Patient 2) “Then I rest a little more in the afternoon. The worst time is usually at 4pm, then it usually starts to get better again but I have to eat a lot of snacks and things.” (Patient 3) “Worry, worry, insecurity, and this applies to both my partner and me, you think one day at a time, you think that this day will be good.”(Relative 6)
Main theme	**Searching for outside assistance to attain safety in the chaos**
Subtheme	**Requesting timely tangible support**
Quotes	“Yes, we get a lot of help, mainly from my mother and my wife’s mother, in taking care of our daughter. So that…well, I couldn’t… I kind of can’t handle a whole day by myself with our daughter now.” (Patient 4) ”Well, I read that in the care guide, so it was very unfortunate. Yes, it said “probably a brain tumour, a primary brain tumour” and I remember yelling at my husband that “oh my God, I have a brain tumour”. So it was very unfortunate that the doctor didn’t have time to inform me before. Yes, it was very unpleasant, very.” (Patient 1) “So there’s something called support for relatives, which you can get if you stop working to take care of your loved one, but it’s 90 days, and it’s probably at the end of life, when there is no hope, but I would like to see that I could be on sick leave to take care of her.[…] but there’s nothing. I’m not allowed sick leave because she … to care for her. Yes, so there are the 90 days, but when they’re over, there’s like nothing left. And I would have wanted that. […] Yes, then we wouldn’t have needed home health care and not the home service.” (Relative 10)
Subtheme	**Needing adapted and available care**
Quotes	“Mm, really. I thought so, that it was… Yes, but you can feel a little stressed, because you have to be on time, and so, and that you: ‘Now I have to follow this program that I’ve been given, two days a week’, and so… Yes, and if it hasn’t worked out, then there hasn’t been any problem like, if I said: ‘I can’t come right now’, they (Rehab training) just said: ‘Yes, well that’s okay, you can start when you feel a little bit better, like that,’ so it’s been really nice not to feel pressured by it [laughs] in that way. That you can do it according to your own ability. And then you also need a bit of pushing, I can feel that sometimes, and I got that too, I think. That sometimes it can be easy to just say ‘No, I’m so sick and I’m so tired and I’m in so much pain, I’m not bothering with this,’ and that’s not good. So there’s been a reasonable mix of understanding and like, ‘No, try to do thirty now.’ So I’m also very happy with that.” (Patient 4) “I have to, like, conserve my energy as well, I still have to rest quite a lot during the days, even though it has got better. So, an entire day can be spent trying to get hold of someone [HCP], and then I’m tired afterward. And then maybe I [laughs] don’t have the energy to do what I have to here at home, because it’s a bit like: Should I take on that struggle now? [to call the HCP] Or should I wait until tomorrow?” (Patient 4) “I know, my husband told you about the waiting time after the X-ray and so on, they could do that because they are supposed to notify earlier, but it is a very long wait, and then it will be a lot of pressure to wait and think about it. You think something is wrong and yes, you invent possible things that could be the cause. So, it’s unnecessary, it’s unnecessary waiting. And as my neurologist told me once a long time ago, “call me in a week and I will find out the pictures right away, so I know.” But the doctor never answered, he was always away or ill or yes, so it hasn’t worked. That thing, I wish they were faster, it’s almost inhumane if people must wait a month for the results.” (Patient 1) “So living in the uncertainty that goes on in three-month cycles until you’re at the next examination, [MRI scan] it’s not… it’s tough. It is. It’s really tough.” [wanted faster response from HCP] (Relative 7) “No, it’s more like you can have digital meetings based on the need and what the patient wants. Dad, he would have wanted that. To have digital meetings and not travel so much, and then maybe they could visit him at home too. That’s what we’ve both thought about. It would save some time and energy for him. Or time for me and energy for him. He does get quite tired after these trips.” (Relative 9) “What do you think is important, if you think about it, that the healthcare staff think about in the meeting with you and your husband? IP: Yes, that’s what they actually do, that they listen carefully and confirm and answer questions and provide the necessary information.” (Relative 8)
Subtheme	**Desiring emotional and existential support**
Quotes	“He’s 33 years old this year, but he has really encouraged me immensely. Yes, and I have a very good relationship with my daughter, and with my husband. We’ve always been able to talk about everything. I haven’t had to say that I’m not scared, or I’ve been able to talk about everything openly. They have supported me.” (Patient 1) “And I understand that this is probably part of … I might be wrong now, but this is probably what has created it … because there is some chaos in healthcare too, in my opinion, it might be this that has caused it, I mean, that they have had to work under difficult conditions.” [the patient describes difficulty to receive emotional support] (Patient 6) “But it’s more those existential questions, [laughs], that are… that I want help with, but I don’t know how I should… I can’t expect a straightforward answer to that, of course, I understand that. Then you might think, wonder: ‘Why me?’, lots of things like that: ‘What had I’…” (Patient 4) “Then, it is a health social worker, I have talked a little with her, and I can call one of the teams, there they have two nurses one can talk to sometimes, like that, when I get panic attacks and want to get up at night, or something…//” (Relative 8) “There will be so much anyway, care contacts as well, and then, I’ve already had to deprioritize myself, that… the spiritual health, so they changed people, so I met a deacon for a period, and she was also good and so, but I would probably have needed help from a more treating contact, constantly, but I sort of had to solve that on my own.” (Relative 8)

**Table 3:** Summary of possible development of support based on the results

Themes	Subthemes	Possible development of support based on the results
Hovering between living in chaos and finding strategies in daily life	Living in uncertainty	Support to manage uncertainty and give more information about: The effect of treatment Survival duration The long and short-term future Fear of worsening symptoms (e.g., tumor-related seizures) Feelings of hopelessness and powerlessness
	Living with cognitive consequences	Support to manage cognitive consequences and educate in how to: Encourage/facilitate activity due to inactivity Support in managing fatigue, impaired executive ability, lack of concentration, memory problems, personality changes Encourage socializing with others
	Living in isolation	Support to manage isolation Discuss why they avoid socializing and possible achievements in relation to social activities Especially support relatives so they can socialize Encourage/facilitate continued work (maybe through digitalization) for both patients and relatives
	Managing the new life situation	Support to manage the new life situation Help to prioritize what they find is most important in life Encourage and facilitate external support to continue with practical activities Help to manage physical and cognitive symptoms Help to find shorter-term goals for days or weeks Encourage personal interests such as traveling or reading Encourage time spent with family and friends Encourage the family to plan and talk about the future Extra support for patients treated with TTF
Searching for outside assistance to attain safety in chaos	Requesting timely tangible support	Facilitate timely tangible support Cooperate for rapid support from both the specialized care team and municipal care due to rapid deterioration Encourage the municipality care to offer quick efforts to increase patients’ sense of security Offer support from the health social worker Support the whole family to reduce the care burden, based on their needs
	Needing adapted and available care	Facilitate adapted and available care Meet the patient/relative as a unique and valuable person Take them seriously, listen to their needs and show encouragement and understanding Avoid appearing stressed since it causes anxiety Individualize the care and provide clear communication based on facts Patients without close relatives might need a different kind of support Adapt information according to how much the patient wishes to know Enable more accessible care and guiding to make it easier to get in touch with the relevant HCP Give clear information about different types of support Extend availability into the evenings, nights, and weekends Avoid waiting time for scheduled doctor’s visits and surgery, which caused concern and fear (e.g., give faster feedback on MRI and treatment decisions) Encourage more optimal technical solutions, especially tools and applications for the patient’s phone, e.g., support for memory and orientation, and to connect aids to existing applications Enable digital care meetings if the patients and relatives wished Video can be easier than telephone since telephone meetings require more concentration Face-to-face meetings are preferred when serious information is being given Involve relatives in conversations with the HCP
	Desiring emotional and existential support	Give emotional and existential support Help the families face their emotional and existential issues Offer a treating psychologist or a health social worker if needed Give relevant hope, faith and coaching Prioritize support around the time of diagnosis and surgery, as well as when complications occur due to treatment or the progression of the disease Encourage emotional support from family and friends Encourage the family to talk about the disease and the future with each other Encourage digital technology in meetings between family and friends if necessary Encourage relatives to network with their own friends

### Hovering between living in chaos and finding strategies in the daily life

The new life situation brought upon by the disease led to chaos for patients and relatives. They lived in uncertainty, were negatively affected by the cognitive symptoms of the patient and became isolated. Finding strategies to manage their new life situation helped them in the experienced chaos.

#### Living in uncertainty

Both patients and relatives felt stressed, worried, and anxiety-ridden by the uncertainty caused by the disease, such as the effects of treatment, fear of worsening symptoms, short- and long-term outlook, and not knowing how long the patient would survive. In other words, their future had collapsed. Both patients and relatives feared tumor-related seizures, with patients afraid of being left alone, falling asleep and not waking up. Relatives also described hopelessness and powerlessness.

#### Living with cognitive consequences

The cognitive consequences of the disease implied inactivity, which affected both patients and relatives. Fatigue, impaired executive ability, and lack of concentration made it difficult for the patients to tackle tasks. Further, they felt that social events made the symptoms worse. Both patients and relatives were affected by the patient’s memory deficits. Not being able to remember what they had learned, the patients could not orientate themselves, causing their relatives to become worried. Some patients experienced behavioral changes, exhibiting signs of apathy, anger and irritability which caused them to feel anxious and guilty about treating their relatives badly.

#### Living in isolation

Due to the symptoms, treatment and the increased risk of infections, several patients and their relatives experienced feelings of isolation, inactivity, and loneliness, leading to increased confinement to their homes and immediate surroundings. Patients often withdrew from social interactions, distancing themselves from family and friends, which further contributed to their sense of isolation and loneliness. These challenges were even more pronounced among relatives, who not only shared the physical and emotional burden but also felt an intensified sense of responsibility, isolation, and loneliness. Patients and relatives expressed a wish to continue working, albeit with a reduced workload. Quitting work isolated them, while continuing their professional activity enhanced feelings of normal everyday life and of “being in a sanctuary”, especially for the relatives. Digitalization facilitated work continuity.

#### Managing the new life situation

Patients adapted to their new life situation by prioritizing important activities despite reduced capacity, managing symptoms, and coping with the inability to drive, with some resorting to cycling or using mopeds. Both patients and relatives adjusted expectations by setting short-term goals and maintaining personal interests. They both emphasized spending time with loved ones and avoiding excessive focus on the disease, while also together planning for the future after death, including completing wills and organizing their affairs for the benefit of their relatives.

A few patients underwent treatment utilizing tumor treating fields (TTF). Enduring adhesive bandages on the skull that generated heat up to 18 h a day was excruciating. They reported feelings of apathy and a decline in overall well-being, expressing that if forced to continue the TTF it was better for them to die. During intervals of treatment cessation, patients found themselves merely awaiting the resumption of the regimen, devoid of any capacity for proactive engagement. They emphasized the necessity for psychological support to navigate through the rigors of the treatment.

### Searching for outside assistance to attain safety in chaos

To feel safer in their chaotic life situation, the patients and their relatives needed concrete, timely support that was adapted to their own needs. It had to be readily available and given in a compassionate way, incorporating emotional and existential support.

#### Requesting timely tangible support

Most patients and relatives requested timely and tangible practical support to make their life situation easier. The rapid progression of the disease required continuous adjustments to changing needs. They especially found municipal support inadequate for handling fast physical, psychological, and cognitive deterioration, relying more on specialized care. Services like cleaning, medical provision, and alarms enhanced safety and independence at home, and it also freed up the relatives. Some patients sought administrative assistance with grant applications and contacted the Swedish Social Insurance Agency. Some relatives expressed financial concerns and called for improved societal support to lighten the burden of the caregiver. Most patients and their relatives described needing support from family and friends. Close family members assisted with daily tasks like cooking and childcare and helped to find solutions to manage their life situation. Some relatives felt responsible for supporting the patient and facilitating access to care, treatment, and aids. They expressed contradictory feelings of both burden and gratefulness, when supporting their loved ones.

#### Needing adapted and available care

All patients and relatives emphasized the importance of adapted and easily available care. They expressed a desire for more accessible care and easier contact with the relevant HCP. They wanted clear communication based on facts and information about different types of support. Some relatives also wanted individualized care for the patient and wished for care availability to be extended to evenings, nights, and weekends. Further, they appreciated when the HCP was able to adapt information according to how much the patient wished to know and they emphasized that patients without close relatives might need a different kind of support.

The long waiting times for scheduled physician visits and examinations caused concern and fear. Several patients and their relatives expressed a desire for faster feedback, particularly regarding MRI results, as the waiting period for these outcomes induced fear and anxiety. Additionally, they sought quicker feedback on treatment decisions and other examinations. Some relatives considered the response time from HCP too lengthy and desired improved technical solutions to improve the response time. Several patients and relatives appreciated the option to choose between physical or digital meetings with HCP. Digital meetings were often more practical, and video calls were easier than telephone calls due to the patients’ reduced concentration caused by the disease. Face-to-face meetings were preferred for more critical information. Involving relatives in conversations with the HCP greatly reassured both patients and relatives. Relatives wished for digital aids for memory and orientation, as well as tools and applications for telephone calls, along with better integration of these aids with existing applications.

#### Desiring emotional and existential support

Many patients and relatives appreciated emotional and existential support from the HCP in terms of their helpfulness and guiding function. Both expressed that they wanted to be met as a unique and valuable person and felt more secure if they were listened to, taken seriously, and met with encouragement and understanding by HCP. Although this was the case more often than not, patients and relatives became worried if HCPs were stressed, if they rushed things, exhibited a tough attitude, or were not up to date with the patient’s health status.

Some patients and relatives requested care from a treating psychologist instead of a health social worker. They wished the HCP could instill more hope and faith and wanted more of a coaching style of support. Some patients and relatives were not interested in participating in support groups, as they believed it would lead to sadness and excessive focus on the disease.

It was stated that the most crucial times for support were around the times of diagnosis and surgery. After surgery, relatives understood that the prognosis was poor and after radiotherapy, they sometimes felt that there was nothing more to do. Increased support and more frequent follow-ups were needed in case complications occurred through treatment or through the progressive nature of the disease.

Emotional support from family and friends was important to the patients and relatives, often strengthening relationships after diagnosis. However, some struggled with discussing the disease and the future, partly because one party wanted to talk about these things, but the other did not. Relatives found such situations particularly burdensome and felt a need to be strong in front of the patient, leaving no room for themselves to be worried or sad. Patients who experienced a lack of support from family and friends felt lonely and isolated. Digital technology offered support when physical meetings were not feasible. Relatives sought support from their own friends, with whom they could freely express themselves and find solace.

The results above indicate possible ways for the developments of individualized support. Table 3 illustrates the components of potential support that are associated with each subtheme.

## Discussion

In this study, patients with glioblastoma and their closest relatives described a time of chaos connected with the disease and a strong desire to find strategies and safety in this chaotic life situation. They experienced a sense of loss of the future and isolation and tried to create and maintain a life under very uncertain conditions. To be able to do so, they needed help from family and friends, but also from HCP. Timely, tangible support, and adapted, readily available care, as well as emotional and existential support, increased their sense of security.

Previous studies have pointed out the risk of isolation and affected relationships among patients with primary malignant brain tumors and their relatives [18, 32]. We found that isolation decreased if patients and relatives stayed active, continued to work, and used digital tools for professional and social communication.

A desire for more accessible care was an important aspect. One problem in this respect was the difficulty in reaching the HCP, requiring a significant investment of time and energy. Both the present study and other studies have highlighted that waiting for the results of the MRI scan examining glioblastoma is anxiety-ridden [33]. Inviting relatives to participate in healthcare meetings, on the other hand, making these accessible to both patients and relatives was found to be a strong positive factor, which is consistent with the findings of Boele et al. [34]. It is likely that relatively easy arrangements like a faster response to MRI follow-up and increased involvement of the closest relatives in patient care will reduce the level of anxiety in both patients and relatives.

Managing uncertainty about the future was difficult, but emotional and existential support, as well as clear information and help at the right time, facilitated the life situation of both patients with glioblastoma and relatives. A previous review focusing on whether support for caregivers could improve psychological distress, mastery, and quality of life showed weak positive results. However, the included articles were of small sample size [35]. Boele et al. [34] have consistently found that a trustful relationship with the care team is crucial. In our study, the information given early on was usually clear, but emergency situations requiring acute surgery, as well as the patient’s state of mind, i.e., the shock, cognitive dysfunction, confusion, and memory problems, made it more difficult to convey information. This is highlighted by a previous study, which concluded that the information provided was insufficient or lacking, and when given, perceived as overwhelming [36]. Importantly, our results showed that the support given by the specialized neuro-oncological team was mostly perceived as positive, whereas support from the municipality was perceived more negatively. This underscores the need for integrated care of patients with glioblastoma spanning from the hospital to the municipality, including palliative care.

Relatives expressed a wish for more optimal technical solutions to make their own and the patients’ everyday lives easier, primarily through application technology for phones to facilitate memory and orientation for the patients. In our increasingly technologically advanced society, healthcare systems still seem to lag behind in adopting modern methods. Prior research highlights the benefits of advanced technology in areas like chronic disease management, mental health, patient education, and empowerment [37]. However, it is also crucial to consider cognitive difficulties in relation to technical literacy. In this context, individual interventions are essential to be able to address the specific needs of each patient. A previous study suggests that patients with brain tumors and their relatives may benefit from interacting with others in similar situations through support groups [16]. Our findings did not suggest a willingness to engage in such groups, primarily due to concerns about excessive emphasis on the disease and negative emotions. Instead, participants sought individually adapted emotional and existential support. This contrasts with a survey involving relatives of patients with primary brain tumors, which did not identify a need for existential support [38]. Support groups can be offered and can be beneficial, but it is individual whether the patient or relatives find them helpful and supportive.

Both our and previous research on patients with brain tumors and their families have shown challenges in accessing support from psychologists, although psychosocial support is especially important in the acute phase of the disease [39]. A review examining care for caregivers in cancer found that psychoeducational and problem-solving/skill-building interventions positively impacted the caregiver’s knowledge base and ability to provide care; further, they could lead to improvements in psychological correlates of burden. Family/couple interventions could also lead to improvements in the functioning of the family or couple as a unit [40].

Our findings are applicable both within clinical practice and as a foundation for future research. To help patients and their relatives manage the uncertainties, clear information about the disease, its prognosis, and treatment is essential. Additionally, support is needed to address the cognitive impacts of the disease, also confirmed by Kirkman et al. [41]. Supporting patients in social interactions and continued employment is another important aspect. Thus, HCP should provide individualized and holistic support throughout the entire disease trajectory, addressing physical, psychological, social, and existential concerns. This comprehensive approach aligns with palliative care goals aimed at enhancing the overall well-being of both patients and their relatives [42, 43]. Moreover, improving collaboration between clinics and hospitals and municipal care settings is crucial. Timely support without organizational hurdles is vital for patients and their relatives. Intervention research that focuses on integrated care throughout the disease progression for both patients and their families will have the potential to enhance the quality of care.

## Conclusion

Glioblastoma affects patients and their relatives, causing cognitive, physical, and psychological challenges that result in strained family dynamics, isolation, and fear of the future. Coping strategies include prioritizing what matters most and setting short-term goals, where support from family, friends, and HCP is crucial. Timely, tangible, and available support is necessary throughout the healthcare journey from diagnosis to end-of-life care. Patients and their relatives require clear information and faster feedback. Individualized care addressing their unique needs, including emotional and existential support, is essential. Our results indicate the importance of integrated, individualized care across the healthcare journey, encompassing specialist neuro-oncological care, municipal support, and palliative care. Individualized support for patients and relatives may improve their sense of security amid the chaos in their life situation.

## Strengths and limitations

The strength of this study lies in its exclusive focus on both patients with IDH-wildtype glioblastoma and their relatives at various disease stages. Studying both provides deeper insight into the changes in life situations brought along by the disease and the support needed from both perspectives. Allowing participants to determine the location of the interview enhances their engagement and ensures that participation is aligned with their own individual circumstances.

A significant limitation is the dropouts, which could imply a skewed bias, with these individuals potentially experiencing a worse life situation and increased need for support. All patients underwent surgery; however, specific details regarding additional treatments were unavailable. Another limitation may be the exclusion of patients with severe cognitive or speech difficulties. However, by including patients with milder impairments and no exclusion criteria for relatives other than adult age, we were still able to obtain a comprehensive understanding of the situation. It can be regarded as a limitation that we did not collect data on ethnicity or employment status, as the Ethics Committee did not approve this part. The cohort, however, included a mixture of native and foreign-born individuals, as well as individuals with different employment statuses.

## Supplementary Information

Below is the link to the electronic supplementary material.

Supplementary file1 (DOCX 18 KB)

## Data Availability

The data are available from the authors upon reasonable request.
