# Supplementary material for: Living with glioblastoma — the need for integrated support based on experiences of chaos, loss of autonomy, and isolation in both patients and their relatives
Source: Support Care Cancer. 2024 Aug 21;32(9):599. doi: 10.1007/s00520-024-08801-y (PMC11339176; doi:10.1007/s00520-024-08801-y)
Supplement: Supplementary file 1 — Supplementary file1 (DOCX 18 KB) [file 520_2024_8801_MOESM1_ESM.docx]

# Supplementary material 1

# Individual interview guide to patients or relatives

If the respondent requires guidance on the topic, supplementary questions (a, b, etc.) will be presented to further explore their understanding.

1. Being diagnosed with a malignant brain tumor can affect one's life in many ways. Can you tell us about when you/your relative was diagnosed?
   1. When did you/your relative fall ill, and what symptoms did you/your relative have at that time?
2. Can you tell me how you feel that your life situation has changed since you got the disease/your relative got the disease?
   1. Have your symptoms changed (your relative’s symptoms) and how does this affect your life?
   2. How has the disease affected your life with those close to you?
3. Can you and your partner or your relatives talk about your life situation, the disease, and the future?
   1. If yes, with whom do you talk, what do you talk about, and what do you think enables you to talk about it?
   2. If no, what do you think prevents you from talking about it?
   3. Are there certain topics you cannot discuss?
   4. Could support to shared conversations for you and your loved one be helpful? Please explain.
4. Do you feel that you and your partner/relative would need increased or another kind of support?
   1. Has it changed during the disease?
   2. What would you like such support to look like?
5. What do you/your relative need help within everyday life?
6. Who/who help you with it and in what way? (Partner/child/parent/others, the tumor team, the oncology clinic, home health care/ASIH, the municipality in the form of e.g. home care, community health/care center, other?
   1. What professions do the people who help you have?
   2. Would you like to have contact with other professions, if so which ones?
7. Do you feel that you and your relative lack support/help in everyday life?
   1. If so, what would you like to receive more support for?
   2. From whom/which would you like more support from (e.g. partner, children, family, friends, the tumor team, the oncology clinic, home care, home health care, palliative care team/AISH, community health)?
   3. In what way would you have liked them to help you and your relatives?
8. What can healthcare/school/care/authorities do to make your family's living situation easier?
9. Do you see any point so far where you have needed more support or different support?
10. What do you think is important for health care professionals to think about in meeting with you and your relatives? What is most important?
11. How could healthcare support strengthen your well-being?
12. What are your thoughts about the future?
    1. Are there worries?
    2. Is there hope?
13. Is there anything you would like to add based on support that you and your loved ones need?
